# Development of a rapid, simple and efficient one-pot cloning method for a reverse genetics system of broad subtypes of influenza A virus

**DOI:** 10.1038/s41598-019-44813-z

**Published:** 2019-06-05

**Authors:** Won-Suk Choi, Ju Hwan Jeong, Khristine Kaith S. Lloren, Su Jeong Ahn, Khristine Joy C. Antigua, Young-il Kim, Young-Jae Si, Yun Hee Baek, Young Ki Choi, Min-Suk Song

**Affiliations:** Department of Microbiology, Chungbuk National University College of Medicine and Medical Research Institute, Cheongju, Republic of Korea

**Keywords:** Influenza virus, PCR-based techniques

## Abstract

The reverse genetics (RG) system of influenza A viruses is well established. However, the conventional sequence-dependent method for cloning influenza genome segments is time-consuming and requires multiple processes (eg. enzyme digestion and ligation) and exhibits low cloning efficiency compared to the sequence-independent cloning method. In this study, we improved influenza genome cloning into the pHW2000 vector for an RG system by incorporating a sequence-independent circular polymerase extension cloning (CPEC) approach which requires only 2 steps (reverse transcription and one-pot CPEC-PCR) and takes about 4 hours before the transformation. The specifically designed viral gene and vector primers used for CPEC-PCR have improved cloning efficiency ranging from 63.6 to 100% based on the results of gene-specific colony PCR which was additionally confirmed by enzyme digestion. We successfully cloned all genes from broad subtypes of influenza A viruses (H1-H12, N1-N9) and rescued by the RG system. Our results demonstrate that this method—one-Pot cloning for influenza A virus—was efficient in terms of required time and cloning rate. In conclusion, the novel cloning method for influenza A virus will contribute to a significant reduction in the time required for genetic studies of emerging influenza viruses.

## Introduction

The influenza A viruses (IAVs) which belong to the Orthomyxoviridae family, consist of eight segments (ie, PB2, PB1, PA, HA, NP, NA, M, and NS)^1^. These viruses have caused pandemics resulting in the death of millions of people worldwide and still pose threats and challenges to public health. Thus, global surveillance and intensive studies to understand the behavior and pathogenicity of these viruses are critical for understanding and controlling the spread of infections. The development of reverse genetics (RG) system which enables the rescue of recombinant influenza A viruses using plasmids became an invaluable tool for studying and understanding viral phenotypes, pathogenesis and/or transmission and to improve the development of anti-viral strategies^2–6^, including new vaccines^7^. Various studies on potential improvements of the RG system have been shown to hasten and simplify the process required for cloning of the eight fragments of the influenza virus into plasmids for influenza virus rescue—a critical component of the RG system^8^. The current process for a sequence-dependent cloning viral genes into an RG vector involves the sequencing, restriction enzyme digestion and ligation of viral genes and vector to generate successful clones, which are onerous and subject to certain limitations such as a lack of appropriate restriction-sites and inefficient enzymatic steps^9–11^. Thus, several groups have recently reported restriction enzyme-free^8,12,13^ and ligation-independent recombination-based approaches for influenza genes cloning^14^ considered sequence-independent cloning approaches, each having their own special characteristics and advantages.

The recent development of a gene cloning method which relies on a mechanism similar to a polymerase chain reaction (PCR) for amplification of DNA sequences, the so-called “Circular Polymerase Extension Cloning” (CPEC), was developed as a simplified sequence-independent cloning technology based entirely on the polymerase extension mechanism^15^. It has notable advantages (eg, high cloning accuracy and efficiency) and uses double-stranded overlapping inserts and vector directly without any treatment such as T4 DNA polymerase treatment or UDG enzymes^15^.

Thus, to accelerate the cloning process for influenza genes, we report rapid cloning of influenza virus genes by applying the CPEC method and improved it by developing a method that can perform simultaneously the amplification of specific influenza gene and CPEC cloning in a one-pot reaction which greatly shortens the cloning process. The new strategy was evaluated and confirmed by cloning the eight influenza genes from a wide range of influenza subtypes (H1-H12 and N1-N9) and rescuing these clones using the RG system.

## Results

### Concept design of the modified pHW2000 vector and PCR primers

**Figure 1:**
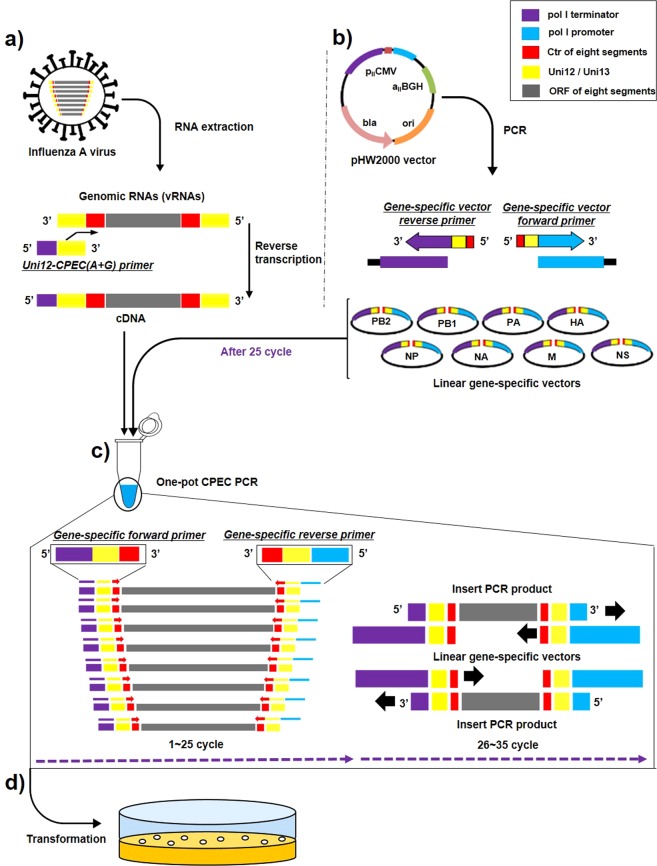
Schematic diagram of the one-pot PCR method for influenza gene cloning. (**a**) First, reverse transcription was performed using the modified uni12 designed primers that are complementary to the 12 conserved nucleotides at the 3′-end of the influenza vRNA to generate complementary DNA (cDNA). (**b**) A gene-specific modified reverse genetics plasmid (pHW2000) was then generated using the designed vector primers to form a linear gene-specific construct for cloning of an individual gene. (**c**) cDNA obtained from reverse transcription and the gel-purified linear vectors are necessary for one-pot PCR. The insert (amplified from cDNA; 1~25 cycles) generated using gene-specific primers results in vector-complementary ends. Following denaturation and annealing [26–35 cycles of Circular Polymerase Extension Cloning (CPEC)], the insert and vector extend using each other as a template to complete a full circle. (**d**) The PCR product with the completely assembled plasmids was used for transformation without further purification.

**Figure 2:**
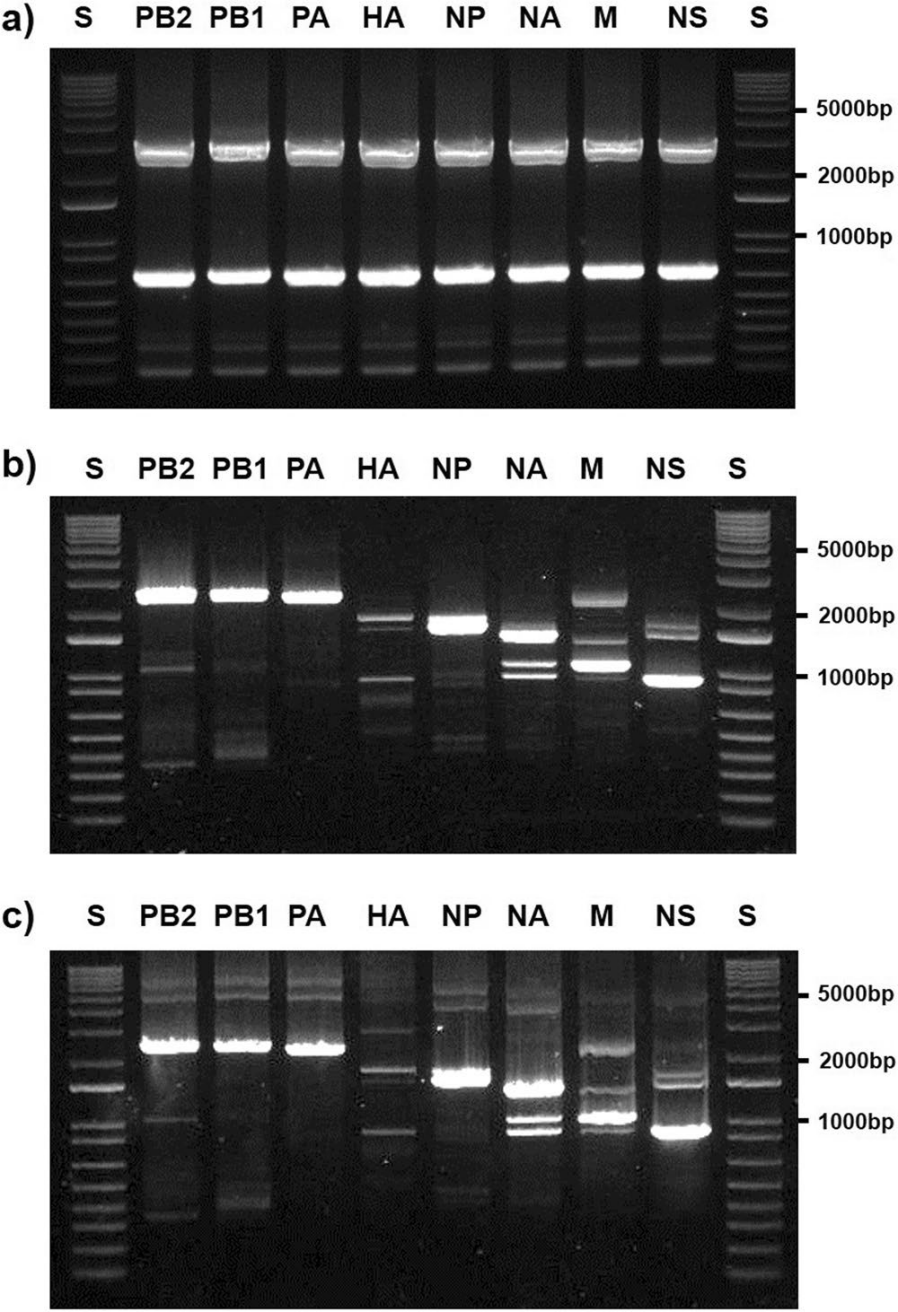
Gel electrophoresis analysis of specific vectors, inserts, and CPEC products. (**a**) Gel analysis of PCR amplicons for modified linear-specific vectors using study-specific primers (as previously described). (**b**) Full-length amplification of all eight genes (PB2, PB1, PA, HA, NP, NA, M, and NS) from cDNA of PR8 to confirm the efficiency of the designed primers. (**c**) Gel electrophoresis analysis of the final assembly products for each influenza segment after 25-cycles of CPEC. All reaction mixtures were separated on a 0.9% agarose gel and visualized using gel red staining. Each gel figures were derived from the complete set of PCR products run in independent gels and were cropped in an appropriate size. S: DNA size ladder (1 kb).

Primers were designed by extending homologous sequences containing Uni 12 or Uni 13 and conserved viral gene-specific sequences of the 8 influenza segments (Table 1) between the polI terminator and polI promotor sequences of the pHW2000 vector (Fig. 1). Using these designed primers and PCR, a linear amplified vector amplicon was obtained. This amplicon was then digested by DpnI and confirmed by gel electrophoresis and viewing under UV light. The results of gel electrophoresis revealed PCR-generated vector amplicons roughly 3 kb in size (Fig. 2a). Furthermore, purified vectors were obtained through gel purification; the eluted vector DNA samples had a concentration of roughly 100~150 ng/µl acquired from 100 μL PCR reactions for each gene-specific vector. The suitability of these vectors for CPEC cloning was confirmed by ensuring that they contained uni12 or 13 sequences and conserved viral gene-specific regions adjacent to the polI terminator or the vector promoter (data not shown).

**Table 1:** Reverse transcription primers and primer sets used for vector and influenza A gene-specific PCR amplification.

Designed gene	Forward Primer (5′ → 3′)		Reverse Primer (5′ → 3′)		Expected size (bp)
Primer for generation of gene-specific vector	pHW2000 - PB2 Universal vector Forward	*AAACGA*ccttgtttctactAATAACCCGGCGGCCCAAAATGCCGACTCG	pHW2000 - PB2 Universal vector Reverse	*GA*cctgctttcgctCCCCCCCAACTTCGGAGGTCGACCAGTACTCCGGTTA	2961 + 8
	pHW2000 - PB1 Universal vector Forward	*AAATG*ccttgtttctactAATAACCCGGCGGCCCAAAATGCCGACTCG	pHW2000 - PB1 Universal vector Reverse	*TG*cctgctttcgctCCCCCCCAACTTCGGAGGTCGACCAGTACTCCGGTTA	2961 + 7
	pHW2000 - PA Universal vector Forward	*AAGTA*ccttgtttctactAATAACCCGGCGGCCCAAAATGCCGACTCG	pHW2000 - PA Universal vector Reverse	*GTA*cctgctttcgctCCCCCCCAACTTCGGAGGTCGACCAGTACTCCGGTTA	2961 + 8
	pHW2000 - NS Universal vector Forward	*AAAACA*cccttgtttctactAATAACCCGGCGGCCCAAAATGCCGACTCG	pHW2000 - HA Universal vector Reverse	*CC*cctgcttttgctCCCCCCCAACTTCGGAGGTCGACCAGTACTCCGGTTA	2961 + 8
	pHW2000 - NP Universal vector Forward	*AAAATAC*ccttgtttctactAATAACCCGGCGGCCCAAAATGCCGACTCG	pHW2000 - NP Universal vector Reverse	*TAC*cctgcttttgctCCCCCCCAACTTCGGAGGTCGACCAGTACTCCGGTTA	2961 + 10
	pHW2000 - NA Universal vector Forward	*AAAAAACT*ccttgtttctactAATAACCCGGCGGCCCAAAATGCCGACTCG	pHW2000 - NA Universal vector Reverse	*ACT*cctgcttttgctCCCCCCCAACTTCGGAGGTCGACCAGTACTCCGGTTA	2961 + 11
	pHW2000 - N3 Universal vector Forward	*AAAAAGCA*ccttgtttctactAATAACCCGGCGGCCCAAAATGCCGACTCG	pHW2000 - N3 Universal vector Reverse	*GCA*cctgcttttgctCCCCCCCAACTTCGGAGGTCGACCAGTACTCCGGTTA	2961 + 11
	pHW2000 - NS Universal vector Forward	*AAAACAC*ccttgtttctactAATAACCCGGCGGCCCAAAATGCCGACTCG	pHW2000 - N6 Universal vector Reverse	*CATTTTCAC*cctgcttttgctCCCCCCCAACTTCGGAGGTCGACCAGTACTCCGGTTA	2961 + 16
	pHW2000 - NS Universal vector Forward	*AAAACAC*ccttgtttctactAATAACCCGGCGGCCCAAAATGCCGACTCG	pHW2000 - N7Universal vector Reverse	*CATTCTCAATCAC*cctgcttttgctCCCCCCCAACTTCGGAGGTCGACCAGTACTCCGGTTA	2961 + 20
	pHW2000 - N9 Universal vector Forward	*AAGAC*ccttgtttctactAATAACCCGGCGGCCCAAAATGCCGACTCG	pHW2000 - N9 Universal vector Reverse	*GAC*cctgcttttgctCCCCCCCAACTTCGGAGGTCGACCAGTACTCCGGTTA	2961 + 8
	pHW2000 - M Universal vector Forward	*AAAAACTA*ccttgtttctactAATAACCCGGCGGCCCAAAATGCCGACTCG	pHW2000 - M Universal vector Reverse	*CTA*cctgcttttgctCCCCCCCAACTTCGGAGGTCGACCAGTACTCCGGTTA	2961 + 11
	pHW2000 - NS Universal vector Forward	*AAAACAC*ccttgtttctactAATAACCCGGCGGCCCAAAATGCCGACTCG	pHW2000 - NS Universal vector Reverse	*CAC*cctgcttttgctCCCCCCCAACTTCGGAGGTCGACCAGTACTCCGGTTA	2961 + 10
Primer for generation of Influenza A virus 8 genes	CPEC - PB2 1 F	AAGTTGGGGGGGagcgaaagcagg*TC*	CPEC - PB2 2341 R	GGTTATTagtagaaacaagg*TCGTTT*	2341 + 19
	CPEC - PB1 1 F	AAGTTGGGGGGGagcgaaagcagg*CA*	CPEC - PB1 2341 R	GGTTATTagtagaaacaagg*CATTT*	2341 + 19
	CPEC - PA 1 F	AAGTTGGGGGGGagcgaaagcagg*TAC*	CPEC - PA 2341 R	GGTTATTagtagaaacaagg*TACTT*	2233 + 19
	CPEC - HA 1 F	AAGTTGGGGGGGagcaaaagcagg*GG*	CPEC - NS 890 R	GGTTATTagtagaaacaagg*GTGTTTT*	1778 + 19
	CPEC - NP 1 F	AAGTTGGGGGGGagcaaaagcagg*GTA*	CPEC – NP 1565 R	GGTTATTagtagaaacaagg*GTATTTTT*	1565 + 19
	CPEC - NA 1 F	AAGTTGGGGGGGagcaaaagcagg*AGT*	CPEC - NA 1413 R	GGTTATTagtagaaacaagg*AGTTTTTT*	1413 + 19
	CPEC - N3 1 F	AAGTTGGGGGGGagcaaaagcagg*TGC*	CPEC - N3 1420 R	GGTTATTagtagaaacaagg*TGCTTTTT*	1420 + 19
	CPEC - N6 1 F	AAGTTGGGGGGGagcaaaagcagg*GTGAAAATG*	CPEC - NS 890 R	GGTTATTagtagaaacaagg*GTGTTTT*	1413 + 19
	N7 1 F(CPEC)(A)	AAGTTGGGGGGGagcaaaagcagg*GTGATTGAGAATG*	CPEC - NS 890 R	GGTTATTagtagaaacaagg*GTGTTTT*	1413 + 19
	N9 1 F(CPEC)(A)	AAGTTGGGGGGGagcaaaagcagg*GTC*	CPEC - N9 1413 R	GGTTATTagtagaaacaagg*GTCTT*	1413 + 19
	M 1 F (CPEC)(A)	AAGTTGGGGGGGagcaaaagcagg*TAG*	CPEC - M 1027 R	GGTTATTagtagaaacaagg*TAGTTTTT*	1027 + 19
	NS 1 F((CPEC)	AAGTTGGGGGGGagcaaaagcagg*GTG*	CPEC - NS 890 R	GGTTATTagtagaaacaagg*GTGTTTT*	890 + 19
Primer for Reverse transcription	Influenza A uni12 -CPEC (G)	CGAAGTTGGGGGGGagcgaaagcagg			
	Influenza A uni12 –CPEC (A)	CGAAGTTGGGGGGGagcaaaagcagg			

The lowercase indicates the 12 or 13 conserved nt in influenza vRNA termini, and the italicized indicates the influenza segment specific sequence. The bold and underlined are derived from pHW2000 regions. The expected size of the PCR amplicons is based on the total length of the genes of A/PR/8/34(H1N1) plus the non-influenza sequences which may differ in the HA, NA and NS for the other influenza A viruses.

To generate cDNA by reverse transcription—a process which enabled the amplification of all eight segments of influenza A viral RNA—two primers were designed to include sequences complementary to the conserved vRNA-termini applicable to all subtypes of Influenza A virus (Table 1)^16–18^. The modified primers also enabled high-amplification efficiency of longer P genes (ie, PB2, PB1, PA) compared to the use of the conventional single uni12 primer (data not shown). Subsequently, to verify the newly designed primers for amplification of viral gene segments, PCR amplicons of the influenza genes were generated using the specifically designed viral gene segment amplifying primers (Table 1) which contain 25–37 nucleotides, homologous with the 5′ and 3′ terminus of the gene specific vectors—a component critical for CPEC cloning. The amplified viral gene segments were confirmed to be the appropriate size (including the newly designed primers) as shown in Fig. 2b.

### Segment amplification using cDNA and integration of CPEC cloning into a one-pot reaction

**Figure 3:**
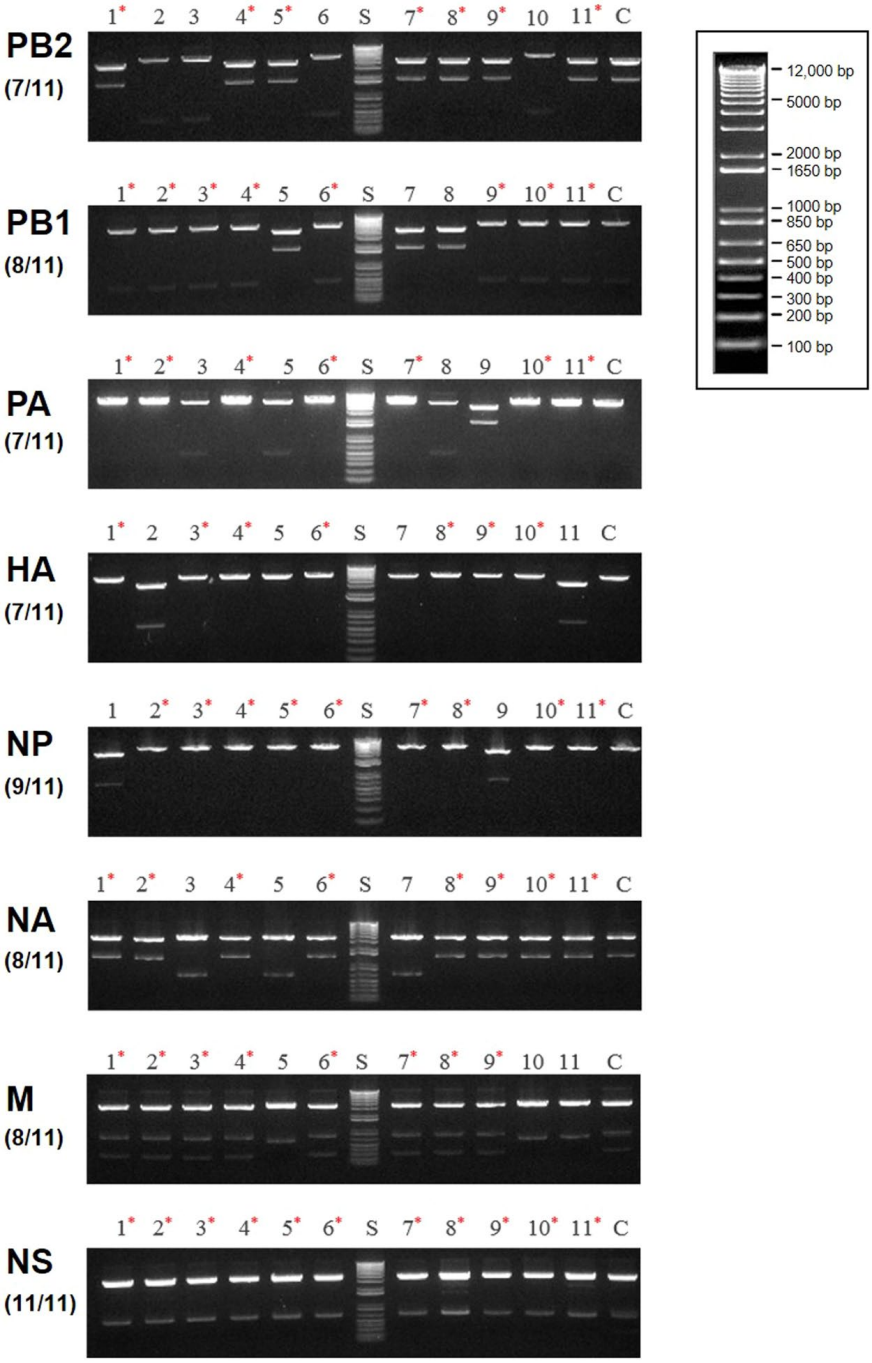
Confirmation by enzyme digestion of PR8-specific genes cloned in the reverse genetic vector pHW2000 using a one-pot CPEC cloning method. NcoI-restriction enzyme analysis of plasmid DNA purified from transformants revealed digestion patterns similar to the positive control. Each gel figures were derived from the complete set of PCR products run in independent gels and were cropped in an appropriate size. The gels were grouped according to the 8 influenza genes (PB2, PB1, PA, HA, NP, NA, M, and NS). S: DNA size ladder (1 kb); 1~11: samples 1 to 11 colonies for each group; C: positive control using specific PR8 plasmid from conventional cloning; *samples with similar digestion patterns to the positive control.

The advantage of our one-pot CPEC strategy for influenza A virus cloning is that it can reduce the multiple steps required for the traditional approach (eg, confirmation and elution of PCR products in an agarose gel, enzyme digestion, and ligation), thereby significantly reducing the time and labor required. To evaluate the effectiveness of this novel method, we firstly cloned the PR8 virus. All eight influenza genes were amplified from the cDNA of PR8—using gene-specific primers—for 25 cycles and the PCR conditions described in the materials and methods section. Without separating the PCR amplicons on an agarose gel and purifying them, 300 ng of specific linear vector previously amplified as described in the materials and methods was added directly to the PCR reaction tube. CPEC cloning was then performed for 10 cycles (Fig. 2c). After assembly of the insert with the vector, the PCR products were directly transformed into competent cells. Each of the steps mentioned above (including RNA extraction) were completed within 4~5 hours. At day 2, 58–98 colonies were obtained for each influenza genes wherein 11 colonies were randomly selected from each plate for colony PCR screening, using the gene-specific primers used for segment amplification. Positive clones were identified by the correct size of each influenza gene and summarized in Table 2. The 11 colonies were also further used to make minipreps and were digested by NcoI enzyme and compared to the positive control (ie, conventionally cloned PR8 plasmids) (Fig. 3). The enzyme digestion of miniprep samples which were seeded from colony-PCR positive samples is optional due to the highly correlated result between PCR and enzyme digestion (Fig. 3). Positive colonies resulted in amplicons and digestion patterns consistent with the positive control (Table 2); results were further confirmed by sequencing. In summary, the percentage for positive clones for each influenza genes were 63.6% (PB2), 72.7% (PB1), 63.6% (PA), 63.6% (HA), 81.8% (NP), 72.7% (NA), 72.7% (M) and 100% (NS), indicating that this novel method is accurate and efficient in terms of the cloning rate despite the significantly reduced processing time and step. Thus, through this developed strategy, we rapidly and efficiently acquired all positive clones for the eight influenza genes from the cDNA of PR8 after using this one-pot CPEC approach.

**Table 2:** Cloning efficiency of the tested one-pot CPEC approach.

Virus name (target gene)^a^	Length^b^ (nt)	UsedcDNA Template^c^ (uL)	Vector PCR amplicon^d^ (ng)	Used Vector	Transformed^e^ (*μ*l)	Colonies picked/Total colonies	Positive colonies confirmed by Colony PCR/Total picked colonies^f^	Positive colonies confirmed by enzyme digestion/Total picked colonies^f^	Percentage of Correlation between Colony PCR and enzyme digestion (%)	Cloning efficiency (%)
PR8 (PB2)	2341	3	300	PB2(CPEC)	5	11/75	7/11	7/11	100	63.6
PR8 (PB1	2341	3	300	PB1(CPEC)	5	11/69	8/11	8/11	100	72.7
PR8 (PA)	2233	3	300	PA(CPEC)	5	11/85	7/11	7/11	100	63.6
PR8 (HA)	1779	3	300	HA(CPEC)	5	11/98	7/11	7/11	100	63.6
PR8 (NP)	1565	3	300	NP(CPEC)	5	11/67	9/11	9/11	100	81.8
PR8 (NA)	1399	3	300	NA(CPEC)	5	11/59	8/11	8/11	100	72.7
PR8 (M)	1027	3	300	M(CPEC)	5	11/68	8/11	8/11	100	72.7
PR8 (NS)	875	3	300	NS(CPEC)	5	11/72	11/11	11/11	100	100

^a^The 8 influenza segments of A/PR/8/34(H1N1) were cloned using the one-pot CPEC approach outlined herein into the cloning vector pHW2000.

^b^Expected size of PCR products after colony PCR.

^c^Amount of cDNA (µl) used as template for one-pot CPEC.

^d^Concentration (ng) of the vector added for CPEC cloning.

^e^Amount of PCR product transformed into competent cells.

^f^Percentage of analyzed clones including an insert of the expected size.

### Cloning of influenza genes and virus rescue of broad subtypes of Influenza A viruses

To further evaluate the applicability of the newly developed cloning method, we applied it to all subtypes of influenza viruses (ie, a total of 15 representative influenza viruses from H1 to H12 and N1 to N9 subtypes) which include (i) human H1N1 influenza virus, (ii) highly pathogenic H5N8 avian influenza virus and (iii) several avian influenza viruses isolated from wild birds (Table 3). As a result, all 8 influenza segments of each virus were successfully cloned (Table 3). To further evaluate the efficiency of the cloned genes when used in the virus rescue, we performed an RG system using all 8 cloned genes of each of the above-mentioned viruses. Each of the individually cloned genes in the genetic background of PR8 viruses, and most of the whole genomes of wild-type viruses were successfully rescued (Table 3). These results confirmed that the method developed for cloning of influenza A genes for an RG study was rapid and highly efficient.

**Table 3:** Various subtypes of influenza virus cloned and rescued in this study.

Virus name	Subtype	Cloning and individual rescue confirmation^a^								Whole gene rescue possibility^b^
		PB2	PB1	PA	HA	NP	NA	M	NS	
A/duck/Korea/463/2014	H1N8	+	+	+	+	+	+	+	+	+
A/duck/Korea/369/2008	H2N3	+	+	+	+	+	+	+	+	+
A/duck/Korea/538/2016	H3N8	+	+	+	+	+	+	+	+	+
A/duck/Korea/137/2006	H4N4	+	+	+	+	+	+	+	+	+
A/duck/Korea/562/2016	H5N3	+	+	+	+	+	+	+	+	−
A/duck/Korea/502/2015	H6N2	+	+	+	+	+	+	+	+	+
A/duck/Korea/557/2016	H7N7	+	+	+	+	+	+	+	+	−
A/duck/Korea/563/2016	H8N6	+	+	+	+	+	+	+	+	+
A/duck/Korea/233/2007	H9N2	+	+	+	+	+	+	+	+	−
A/duck/Korea/530/2016	H10N4	+	+	+	+	+	+	+	+	−
A/duck/Korea/552/2016	H11N9	+	+	+	+	+	+	+	+	+
A/duck/Korea/373/2008	H12N5	+	+	+	+	+	+	+	+	+
A/Puerto Rico/8/1934	H1N1	+	+	+	+	+	+	+	+	+
A/Korea/CNH1/2016	H1N1	+	+	+	+	+	+	+	+	+
A/Environment/Korea/W468/2015	H5N8	+	+	+	+	+	+	+	+	+

^a^+ indicates a successful virus rescue for specified viruses using one plasmid made in this study with the seven other PR8 plasmids.

^b^− indicates an unsuccessful rescue of the virus using eight plasmids of the specified virus.

## Discussion

**Figure 4:**
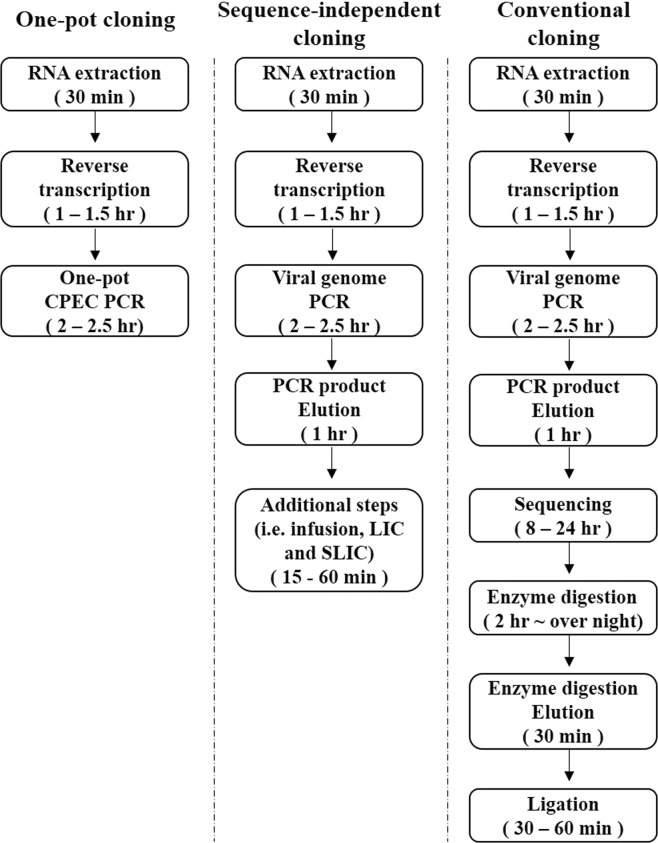
Illustration of streamlined schemes of various cloning methods for reverse genetics systems of influenza viruses. The illustrations begin with RNA extraction; the duration of the cloning processes for each method are represented The sequencing step added in the conventional method during the cloning procedure was done to the verification of the existence of restriction enzyme digestion site in the insert gene. Infusion, a ligation method in the sequence-independent cloning^20^; LIC, Ligation Independent Cloning^21^; SLIC, Sequence and Ligation Independent Cloning^22^.

In order to simplify the process and reduce the time required for cloning methods used to: (i) analyze the molecular characterization of influenza viruses and (ii) produce vaccines and recombinant viruses, we modified the traditional approach and cloned 8 influenza A virus gene segments into the vector (pHW2000) using the primers described above. The whole process involves a single PCR process and transformation after reverse transcription of the viral RNA. Therefore, sequencing, enzyme digestion, and ligation—steps required for traditional sequence-dependent cloning methods—are omitted, and cloning time is greatly reduced (Fig. 4).

Generally, for the insertion of PCR amplicons into a linear plasmid vector using most cloning methods, the insert and the vector should share overlapping homologous terminuses^15^. In contrast to other sequence-independent cloning methods (eg, LIC, UDG-based methods, SLIC and PIPE), the CPEC method uses double-stranded overlapping inserts and vector directly without any treatment^15^, making it more convenient.

In the first step of the one-pot reaction (ie, amplification of the insert from cDNA), only a light band of the correct size was visible upon gel electrophoresis of the PCR product (data not shown), however, positive clones were obtained after the subsequent CPEC reaction (ie, addition of the vector). This suggests a highly efficient method requiring only a small amount of insert to obtain positive clones. Furthermore, the elimination of gel purification after insert amplification is another advantage of this novel approach as it prevents the possible loss of PCR amplicons during extraction from agarose gels, particularly when the abundance of amplicons is low. In this regard, the cloning efficiency in traditional method was low when tested in comparison to that of the One-pot cloning using equal viral gene segment (Supplementary Fig. S1 and Table S1). In addition, due to the limited use of digestive enzymes such as BsmBI and BsaI^19^, the increasing number of digestive cuts in viral gene insert consequently decrease the rate of cloning efficiency (Supplementary Fig. S1 and Table S1). However, the developed one-pot method has successfully cloned these genes, suggesting a higher efficiency and improved method of cloning for such genes.

We designed our CPEC based on the results of previous studies which revealed that 10 cycles of CPEC was sufficient for cloning^15^, thus keeping the number of PCR cycles to a minimum. Additionally, it is not recommended to have too many cycles for the CPEC step as concatemers may form^15^. Since CPEC is not an amplification process and does not accumulate mutations, it was reported that a smaller number of cycles (ie, one five minute denature-annealing-extension cycle) is sufficient for optimal single-gene cloning; 5–25 cycles were recommended for multi-fragment cloning^15^. The PCR polymerases used in the reactions were also considered. In this study, we have used the Phusion DNA polymerase, an enzyme with high fidelity and processivity that had been shown to be highly efficient for the demanding task of CPEC-mediated cloning^15^.

This study also demonstrated the efficiency in an RG system using clones produced by this one-pot cloning method. The method was also applied to clone the gene segments of various influenza A viruses. All plasmids cloned by this method were individually rescued in the backbone of PR8 in an efficient manner. However, even though some representative subtypes of avian viruses (ie, H5N3, H7N7, H9N2, and H10N4) were not successfully rescued using their whole genome—possibly due to the low viral replicability of these viruses in a human cell line (293 T)—the majority of the subtypes of avian and human viruses tested could be successfully rescued using the novel one-pot cloning approach described here. Thus, the method is applicable to the cloning of various subtypes of influenza A viruses. Furthermore, although the pHW2000 vector as a virus rescue platform was specifically used in the current study, the method could also be applicable for the other vectors for influenza rescue system by slight modification of the vector and insert primers based on the sequence termini of the target vector, thus, suggesting a high applicability of the method for cloning of influenza A virus rescue system.

In comparison with other methods for cloning influenza A genes for RG, our newly developed method was confirmed to be a simple, rapid and efficient cloning process with utility in conjunction with an RG system. Some recent similar papers also reported on newly developed approaches for cloning influenza genes with their unique advantages and characteristics (eg, restriction enzyme and ligation-free processes)^12,13,16^. Each of these appears to be improvements over the traditional method for cloning influenza genes. However, neither of these approaches leverage a one-pot reaction involving amplification of influenza genes and integration of the CPEC method to assemble the insert with the vector to generate a product that can be used for transformation in reduced time. Thus, the novel method described herein eliminates the ligation process, gel purification of amplified inserts and the use of restriction enzymes, thereby accelerating the cloning process and offering additional advantages (eg, high efficiency, convenience and cost-effectiveness).

## Materials and Methods

### Viruses and cells

Madin-Darby canine kidney (MDCK) cells were maintained in Eagle’s minimal essential medium (EMEM; Lonza, Allendale, NJ) containing 5% fetal bovine serum (FBS), vitamins (Gibco) and 1% antibiotics (penicillin/streptomycin, Gibco-Invitrogen). Human embryonic kidney (293 T) cells were maintained in Dulbecco’s modified Eagle’s medium (DMEM; Gibco-Invitrogen, Carlsbad, CA) containing 10% FBS and 1% antibiotics. Both cell types were incubated at 37 °C in 5% CO_2_. A wide range of influenza viruses (avian and human isolates) were used in this study, including: (i) A/duck/Korea/436/2014 (H1N8) (W436), (ii) A/duck/Korea/369/2008 (H2N3) (W369), (iii) A/duck/Korea/538/2016 (H3N8) (W538), (iv) A/duck/Korea/137/2006 (H4N4) (W137), (v) A/duck/Korea/562/2016 (H5N3) (W562), (vi) A/duck/Korea/502/2015 (H6N2) (W502), (vii) A/duck/Korea/557/2016 (H7N7) (W557), (viii) A/duck/Korea/563/2016 (H8N6) (W563), (ix) A/duck/Korea/233/2007 (H9N2) (W233), (x) A/duck/Korea/530/2016 (H10N4) (W530), (xi) A/duck/Korea/552/2016 (H11N9) (W552), (xii) A/duck/Korea/373/2008 (H12N5) (W373), (xiii) A/Environment/Korea/W468/2015 (H5N8) (W468), and (xiv) A/Korea/CNH1/2016 (H1N1) (CNH1). In addition, 8 genes of A/Puerto Rico/8/34 (H1N1) (PR8) virus were also used as genetic backbone for the individual rescue of the plasmids from abovementioned viruses to verify that the individual clones of the viruses were properly working. The viruses were propagated in specific pathogen–free (SPF) 10-day-old embryonated chicken eggs and stored at −80 °C until use. Viral RNA of propagated viruses were extracted for cloning by using QIamp Viral RNA Kit (Qiagen Hilden, Germany) following manufacturer’s guidelines. HPAI H5N8 (W468) virus experiments were done in biosafety level three (BSL3+) laboratory approved by the Korea Center for Diseases Control.

### Primer design

The specific primers designed to amplify influenza A genes and modified vectors for this novel cloning approach are presented in Table 1. Due to the highly conserved sequence moiety in 5′ and 3′ non-translational region (NTR) of the influenza A viral gene segments, primers for the amplification of segment-specific vectors were designed with extending segment-specific NTR regions to each gene-segments in order to increase the cloning specificity. Forward vector-specific primers were designed with elongated 5′-ends containing conserved 2–8 viral gene-specific nucleotides (nts) and the uni13 sequence (CCTGCTTTCGC) adjacent to the 30 nucleotides in polymerase I promoter sequence of the pHW2000 plasmid. Reverse primers contain the reverse complement of the conserved viral gene specific (2–13 nts) and the uni12 sequence (AGCGAAAGCAGG or AGCAAAAGCAGG) adjacent to the 37 nucleotides in a terminator sequence of the pHW2000 plasmid (Table 1). For amplification of the influenza A genes, we used gene-specific primers described previously^19^ but with modifications (Table 1) by adding complementary sequences (AAGTTGGGGGGG and GGTTATT) to the forward and reverse primers, respectively to generate a sequence overlapping with the vector. The primers associated with the NA gene are further subdivided according to subtype (for N1, N2, N4, N5, and N8; for N3; for N6, N7 and for N9)^19^. The primers used in the reverse transcription step were designed by extending the vector sequence (CGAAGTTGGGGGGG) with the uni12 (G and A) sequence (Table 1).

### Preparation of a gene-specific vector

To generate a linearized gene-specific vector applicable to all subtypes of influenza A virus, we performed PCR using the primers described above and in Table 1. The PCR reaction was performed using Phusion high-fidelity DNA polymerase (New England BioLabs, Frankfurt am Main, Germany) and a mixture containing 20 µl of 5x HF buffer, 10 µl of 2 mM dNTP, 2 µl of 50 mM MgCl_2_, 4 µl of 5 pmol primer (each), 100 ng of empty pHW2000 plasmid DNA which is digested and linearized by KpnI enzyme, 2 µl of Phusion polymerase, and distilled water (DW) for a total reaction volume of 100 µl. The PCR conditions are as follows: one cycle of 98 °C for 30 s (initial denaturation) followed by 35 cycles of 98 °C for 10 seconds, 58 °C for 30 seconds and 72 °C for 90 seconds, and a final elongation of 72 °C for 7 minutes. PCR products were digested using DpnI to get remove methylated DNA (2 µl enzyme in a total reaction of 100 µl incubated at 37 °C for 2 hours). The DpnI-digested PCR amplicons were purified with QIAquick® Gel Extraction Kit (Quiagen, Valencia CA) according to the manufacturer’s instructions.

### Preparation of viral cDNA

The viral RNAs from avian and human influenza viruses were extracted using QIamp Viral RNA Kit as previously described. For cDNA synthesis, reverse transcription was performed using a 6:4 ratio mixture of the two modified primers Uni12-CPEC (G) and Uni12-CPEC (A) (Table 1). Briefly, RT-PCR included a reaction mixture containing 37 µl of vRNAs, 5 µl of 2 mM dNTP and 2 µl of RT primer mixtures (each 5pmol/µl): (i) annealed at 72 °C for 5 minutes and (ii) quickly transferred to ice for cooling before adding 5 µl of 10x buffer and 1 µl of M-MLV Reverse Transcriptase to a total reaction volume of 50 µl and (iii) incubation at 37 °C for 1 hour. The reaction was halted by enzyme inactivation (72 °C for 10 minutes).

### One-pot CPEC cloning

Gene-specific amplification and CPEC cloning were performed simultaneously in a one-pot PCR reaction. The PCR amplification mixture contained 6 µl of cDNA obtained from reverse transcription described above, 10 µl of 5x HF buffer, 5 µl of 2 mM dNTP, 1 µl of 50 mM MgCl_2_, 4 µl of 5 pmol gene-specific primer set (2 µl each), 1 µl of Phusion Polymerase, and DW to a total reaction volume of 50 µl. After the initial denaturation step (98 °C for 30 seconds), PCR conditions were 25 cycles of: (i) denaturation (98 °C for 10 seconds), (ii) annealing (58 °C for 30 seconds), and extension step at 72 °C for 90 seconds. Next, 300 ng of the gel-purified gene-specific linear vector was added to the reaction and PCR was continued for an additional 10 cycles. The reaction was halted with a final extension (5 minutes at 72 °C). We transformed 5 µl of the CPEC-PCR product into 50 µl of competent cells by heat-shock and plated all on ampicillin plates. The remaining of CPEC-PCR product (45 µl) can be used to analyze viral genome sequence (Fig. 2c), when the viral genome sequence is needed. Random independent colonies were selected and used as a template for single-colony PCR using gene-specific primers to verify the presence of the correct insert. The confirmed colonies were further seeded appropriately and were prepared for plasmid DNA purification by miniprep or midiprep.

### Virus rescue by transfection

The 8 gene-containing plasmids of each of the viral strains used in this study cloned using the one-pot CPEC cloning method described here were used in the genetic reverse system to generate recombinant viruses. Briefly, 1 µg of each of the eight segment plasmids (PB2, PB1, PA, HA, NP, NA, M, NS) were mixed with 182 µl of Opti-MEM medium followed by addition of 18 µl of TransIT®-LT1 Transfection Reagent (Mirus Bio LLC, Madison, WI). The mixture was incubated at room temperature for 45 minutes. 800 µl of Opti-MEM was added and mixed, and then the mixture was added onto the co-cultured MDCK and 293 T cells prepared in 6-well plates. After 18 hours of transfection, the transfection media was replaced with 1 ml of fresh Opti-MEM containing antibiotics and further incubated for 24 hours. 1 ml of Opti-MEM containing 1 µg/ml TPCK-Trypsin was added and incubated further for 48 hours. At day 5 post-transfection, the transfection supernatant samples were injected into 10-day-old SPF embryonated chicken eggs and incubated for 48 to 72 hours. The allantoic fluids were harvested and confirmed for successful rescue of the virus by hemagglutination test and sequences confirmed by RT-PCR. Two additional blind passages were performed when the desired RG-virus was not detected in the first egg passage. Furthermore, two more rescue procedures were also performed when the initial rescue was not successful.

## Supplementary information

Supplementary Figure S1, Supplementary Table S1

## References

[1] Bouvier NM, Palese P (2008). The biology of influenza viruses. Vaccine.

[2] Sedova, E. *et al*. Recombinant influenza vaccines. *Acta Naturae (англоязычная версия)* **4** (2012). PMC3548171 23346377

[3] Neumann G (1999). Generation of influenza A viruses entirely from cloned cDNAs. Proceedings of the National Academy of Sciences.

[4] Neumann G, Ozawa M, Kawaoka Y (2012). Reverse genetics of influenza viruses. Methods Mol Biol.

[5] Lee CW (2014). Reverse genetics of influenza virus. Methods in molecular biology (Clifton, N. J.).

[6] Fodor E (1999). Rescue of influenza A virus from recombinant DNA. Journal of virology.

[7] Nogales Aitor, Martínez-Sobrido Luis (2016). Reverse Genetics Approaches for the Development of Influenza Vaccines. International Journal of Molecular Sciences.

[8] Shao H (2015). An efficient and rapid influenza gene cloning strategy for reverse genetics system. Journal of virological methods.

[9] Klock HE, Koesema EJ, Knuth MW, Lesley SA (2008). Combining the polymerase incomplete primer extension method for cloning and mutagenesis with microscreening to accelerate structural genomics efforts. Proteins: Structure, Function, and Bioinformatics.

[10] Stevenson J, Krycer JR, Phan L, Brown AJ (2013). A practical comparison of ligation-independent cloning techniques. PloS one.

[11] Li MZ, Elledge SJ (2007). Harnessing homologous recombination *in vitro* to generate recombinant DNA via SLIC. Nature methods.

[12] Stech J (2008). Rapid and reliable universal cloning of influenza A virus genes by target-primed plasmid amplification. Nucleic acids research.

[13] Wang S (2008). Simplified recombinational approach for influenza A virus reverse genetics. Journal of virological methods.

[14] Zhou B (2011). Reverse genetics plasmid for cloning unstable influenza A virus gene segments. Journal of virological methods.

[15] Quan J, Tian J (2009). Circular polymerase extension cloning of complex gene libraries and pathways. PloS one.

[16] Zhou B (2009). Single-reaction genomic amplification accelerates sequencing and vaccine production for classical and Swine origin human influenza a viruses. Journal of virology.

[17] de Wit E (2007). Rapid sequencing of the non-coding regions of influenza A virus. Journal of virological methods.

[18] Lee KH, Seong BL (1998). The position 4 nucleotide at the 3′ end of the influenza virus neuraminidase vRNA is involved in temporal regulation of transcription and replication of neuraminidase RNAs and affects the repertoire of influenza virus surface antigens. The Journal of general virology.

[19] Hoffmann E, Stech J, Guan Y, Webster RG, Perez DR (2001). Universal primer set for the full-length amplification of all influenza A viruses. Archives of virology.

[20] Zhu B, Cai G, Hall EO, Freeman GJ (2007). In-Fusion™ assembly: seamless engineering of multidomain fusion proteins, modular vectors, and mutations. Biotechniques.

[21] Aslanidis C, De Jong PJ (1990). Ligation-independent cloning of PCR products (LIC-PCR). Nucleic acids research.

[22] Li, M. Z. & Elledge, S. J. In Ge*ne* Synthes*is*: Met*hods and Protocols* (ed Jean Peccoud) 51–59 (Humana Press, 2012).

