# Supplementary material for: Development of a rapid, simple and efficient one-pot cloning method for a reverse genetics system of broad subtypes of influenza A virus
Source: Sci Rep. 2019 Jun 5;9:8318. doi: 10.1038/s41598-019-44813-z (PMC6549168; doi:10.1038/s41598-019-44813-z)
Supplement: Supplementary file 1 — Supplementary Figure S1, Supplementary Table S1 [file 41598_2019_44813_MOESM1_ESM.docx]

**Development of a rapid, simple and efficient one-pot cloning method for a reverse genetics system of broad subtypes of influenza A virus**

Won-Suk Choi^1,†^, Ju Hwan Jeong^1,†^, Khristine Kaith S. Lloren^1^, Su Jeong Ahn^1^, Khristine Joy C. Antigua^1^, Young-il Kim^1^, Young-Jae Si^1^, Yun Hee Baek^1^, Young Ki Choi^1,*^, Min-Suk Song^1,*^

^1^Department of Microbiology, Chungbuk National University College of Medicine and Medical Research Institute, Cheongju, Republic of Korea.

^*^Corresponding authors, contact at [songminsuk@chungbuk.ac.kr](mailto:songminsuk@chungbuk.ac.kr)

**SUPPLEMENTARY INFORMATION**

**Supplementary Figure S1;**

**Supplementary Table S1.**


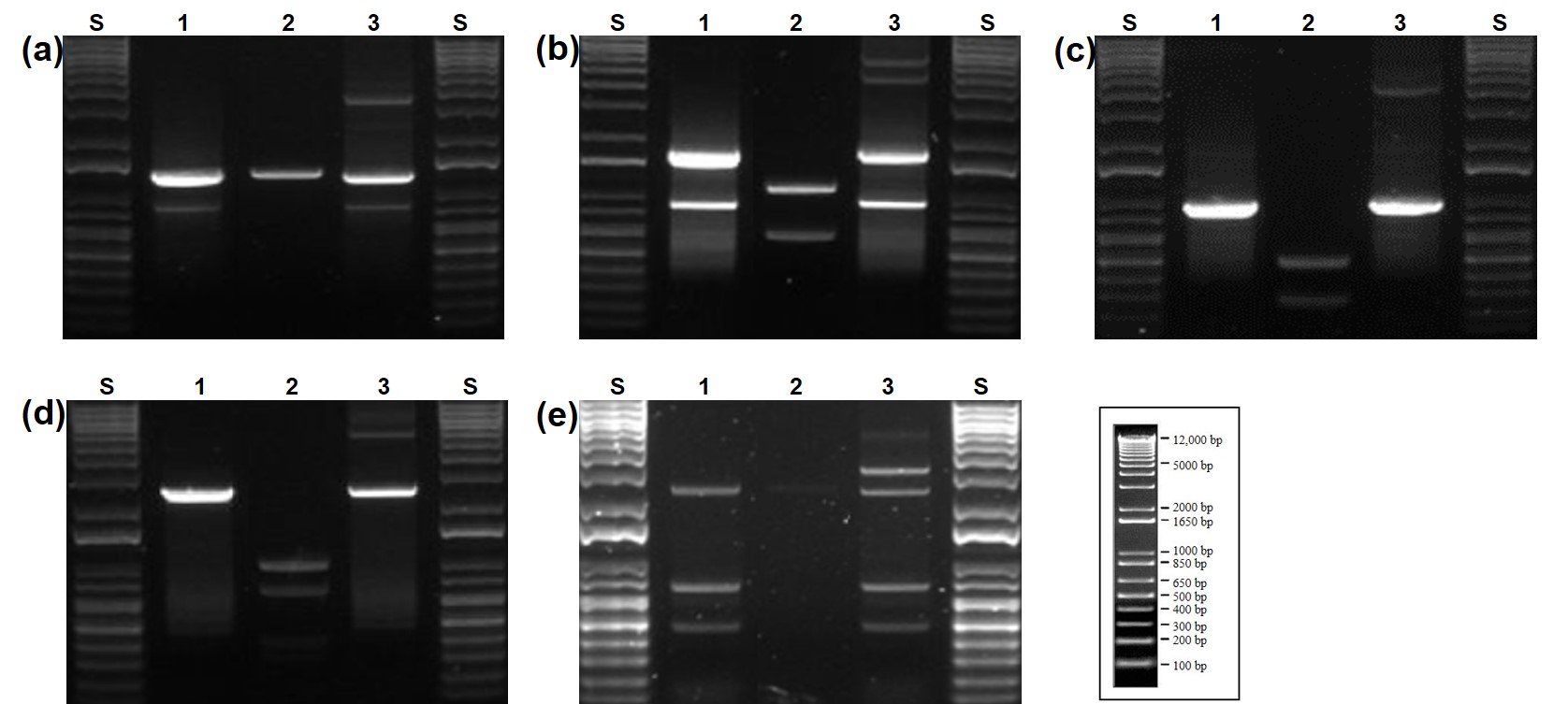


**Supplementary Figure S1. Gel electrophoresis analysis of segments with a variety of restriction enzyme cuts and low concentration of PCR-products**. Results of pH1N1 virus genes with their respective restriction enzyme cut sites (a) NA gene at 0 site; (b) NP gene at 946bp; (c) NS gene at 422/628bps; (d) PA gene at 243/1161/1533bps; and (e) PB1 gene at 0 site but the PCR-product concentration of the target gene is low after RT-PCR. All reaction samples in each segment were separated using 0.9% agarose with gel red staining. 1: RT-PCR result using conventional universal primer for cloning of traditional influenza virus cloning. 2: the result of restriction enzyme digestion of the purified PCR products after RT PCR with each restriction enzyme (state before clean-up or elution). 3: the result of the PCR product after one-pot CPEC cloning. S: DNA size ladder (1 kb).

**Supplementary Table S1.** Comparison of cloning efficiency between traditional influenza cloning method and One-pot CPEC method**.**

| **Virus name (target gene)^a^** | **Restriction**  **enzyme^d^** | **Number of enzyme cut site** | **Positive colony / Total Picked colony^f^** | |  | **Cloning efficiency (%)^f^** | |
| --- | --- | --- | --- | --- | --- | --- | --- |
|  |  |  | **Traditional influenza cloning method** | **One-pot CPEC method** |  | **Traditional influenza cloning method** | **One-pot CPEC method** |
| pH1N1^b^ (NA) | BsaI | 0 | 7/11 | 8/11 |  | 63.6 | 72.7 |
| pH1N1 (NP) | BsmBI | 1 | 5/11 | 8/11 |  | 45.4 | 72.7 |
| pH1N1 (NS) | BsaI | 2 | 6/11 | 10/11 |  | 54.5 | 90.9 |
| pH1N1 (PA) | BsaI | 3 | 2/11 | 6/11 |  | 18.1 | 54.5 |
| Avian influenza virus (PB1)^c^ | BsmBI | 0 | 0/11 | 5/11 |  | 0.0 | 45.4 |

^a^ Segments of influenza viruses with a variety of restriction enzyme cuts were cloned using traditional influenza virus cloning method and the one-pot CPEC

method into the cloning vector pHW2000.

^b^ pH1N1 is A/California/04/2009 (H1N1).

^c^ PB1 segment of A/Environment/Korea/W468/2015 (H5N8) has a low concentration of PCR product after RT-PCR.

^d^ The type of restriction enzyme used in the traditional influenza virus cloning method.

^f^ Result of analyzed clones including an insert of the expected size using colony PCR and NcoI enzyme digestion.

**Supplementary Methods**

*Preparation of linearized vector and insert for Traditional influenza gene cloning*

The linearized vector and influenza viral gene insert were prepared as previously reported[^1^](#_ENREF_1). Briefly, pHW2000 vector was linearized by restriction enzyme digestion using BsmBI and then, the BsmBI-digested vector was further processed by KpnI enzyme digestion to mitigate the self-ligation. The BsmBI-KpnI-digested pHW2000 vector was purified using QIAquick® Gel Extraction Kit (Qiagen, Valencia CA) according to the manufacturer’s instructions. The viral RNA of A/California/04/2009 (H1N1) (CA04) and A/Environment/Korea/W468/2015 (H5N8) (W468) was extracted with QIAamp® Viral RNA Kit according to the manufacturer’s instructions. For cDNA synthesis, Reverse transcription was performed using uni12 universal primer followed by PCR using viral gene-specific universal primers that contains BsmBI or BsaI enzyme sites[^1^](#_ENREF_1). The PCR Amplification Mixture was prepared with 3μL of cDNA, 10μL of 5x HF buffer, 5μL of 2mM dNTP, 1μL of 50mM MgCl2, 4μL of 5pmol universal primers (each 2μL), 1μL of Phusion polymerase and 26μL of D.W. The PCR procedure proceeded as follows: 1) Start of initial denaturation step for 30 seconds at 98°C. 2) At 98°C, degeneration step proceeded for 10 seconds, annealing at 58°C for 30 seconds, and extension at 72°C for 90 seconds, and then repeat of the whole process for 35 cycles. 3) Proceed the last extension at 72°C for 5 minutes. In the PCR product, the target gene was purified using the QIAquick® Gel Extraction Kit (Qiagen, Valencia CA). Purified-gene PCR products were digested with BsmBI or BsaI restriction enzymes and purified using the QIAquick® Gel Extraction Kit (Qiagen, Valencia CA).

*Traditional influenza cloning*

To ligate vectors and segments, the purified digested-gene PCR product and the linearized pHW2000 vector were combined in 1:3 ratios, and the same volume of T4 ligase was added and incubated at 16 °C for 30 minutes (If gene have enzyme cutting site, add the vector after 5 minutes). 5μL of ligation samples were transformed in 50 μL of competent cells and heat-shocked and plated all on ampicillin plates. The colonies were randomly selected and determined if the correct gene was cloned by enzyme digestion using NcoI after mini-prep plasmid purification.

**Reference**

1 Hoffmann, E., Stech, J., Guan, Y., Webster, R. G. & Perez, D. R. Universal primer set for the full-length amplification of all influenza A viruses. *Archives of Virology* **146**, 2275-2289, doi:10.1007/s007050170002 (2001).
